# Intention Recognition With ProbLog

**DOI:** 10.3389/frai.2022.806262

**Published:** 2022-04-26

**Authors:** Gary B. Smith, Vaishak Belle, Ronald P. A. Petrick

**Affiliations:** ^1^Edinburgh Centre for Robotics, Edinburgh, United Kingdom; ^2^School of Informatics, University of Edinburgh, Edinburgh, United Kingdom; ^3^Alan Turing Institute, London, United Kingdom; ^4^Department of Computer Science, Heriot-Watt University, Edinburgh, United Kingdom

**Keywords:** intention recognition, goal recognition, probabilistic logic programming, smart home, assisted living at home

## Abstract

In many scenarios where robots or autonomous systems may be deployed, the capacity to infer and reason about the intentions of other agents can improve the performance or utility of the system. For example, a smart home or assisted living facility is better able to select assistive services to deploy if it understands the goals of the occupants in advance. In this article, we present a framework for reasoning about intentions using probabilistic logic programming. We employ ProbLog, a probabilistic extension to Prolog, to infer the most probable intention given observations of the actions of the agent and sensor readings of important aspects of the environment. We evaluated our model on a domain modeling a smart home. The model achieved 0.75 accuracy at full observability. The model was robust to reduced observability.

## 1. Introduction

From autonomous vehicles to hotel check-in robots, autonomous systems are progressively more present in human environments. One of the most unpredictable and difficult factors of the environment for autonomous systems to manage is humans themselves. Humans can be potential obstacles, collaborative partners, customers in need of assistance, or patients in need of care. To navigate these complex interactions effectively and safely, it is important for autonomous systems to be able to reason about the intentions of human agents (Sadri, 2011). Understanding the intentions of humans aids autonomous systems in predicting the future actions of the human agents they interact with. This capability can enable an autonomous system to more effectively plan actions and react to humans in the environment. For example, knowing that a child's goal is to catch a ball rolling toward a carriageway enables an autonomous vehicle to predict that the child may step into the road and thus plan evasive actions.

**Figure 1:**
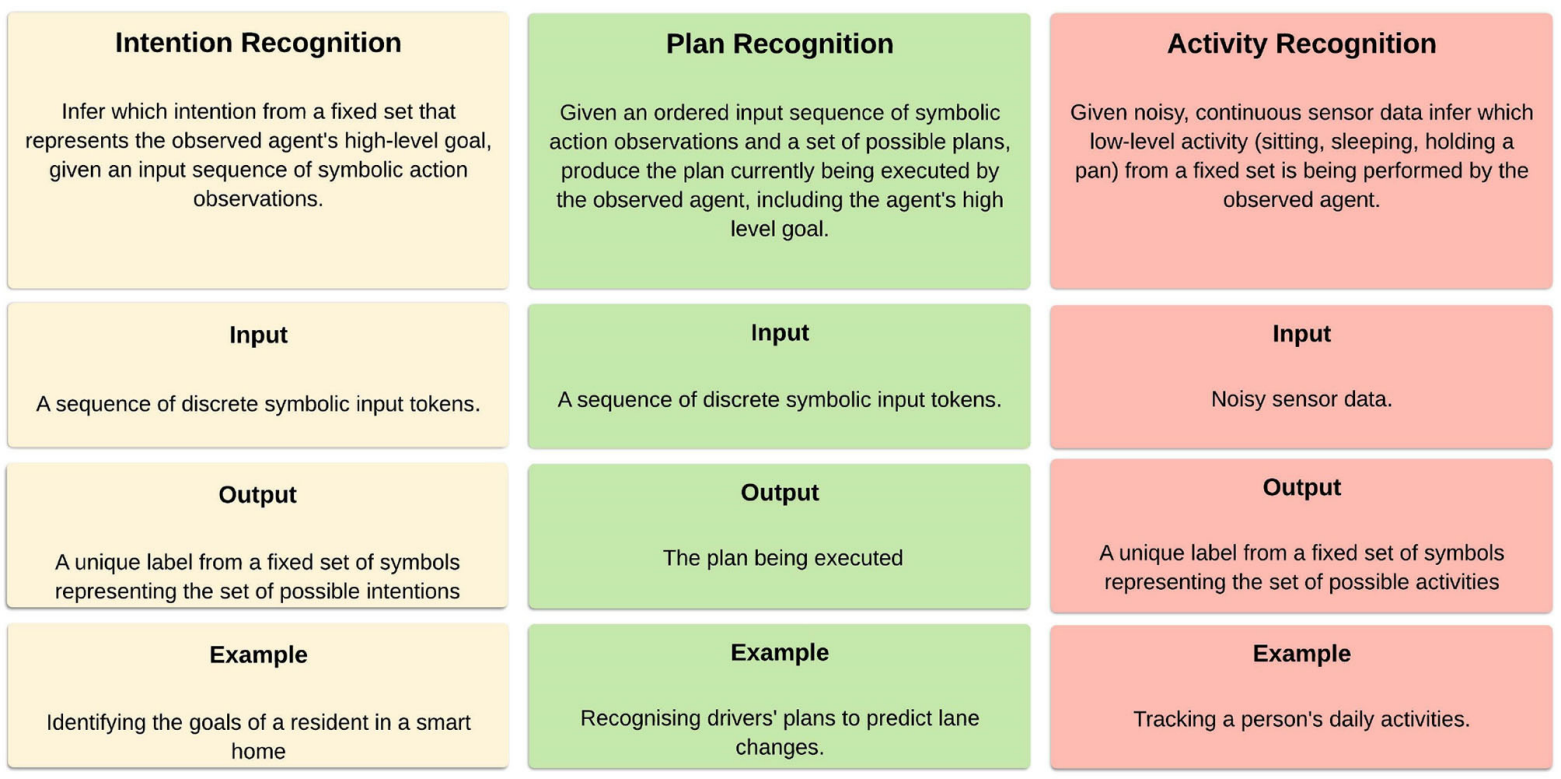
Comparing intention, plan, and activity recognition.

Intention recognition is the process of inferring the intentions or goals of an agent by analyzing their behavior (Sadri, 2011; Mirsky et al., 2021). The terms *intention recognition* and *goal recognition* can be used interchangeably. Intention recognition is related to plan recognition, the process of inferring not only the intention of an agent, but also the sequence of actions the agent will use to achieve that intention (Sadri, 2011). A further related recognition problem is activity recognition. Activity recognition is the task of inferring the activity being performed by an observed agent in a given time window from noisy sensor data (Mirsky et al., 2021). These three recognition problems are compared in Figure 1. Though all of these recognition problems are closely related, in this work we focus exclusively on intention recognition.

A motivating scenario in which intention recognition is particularly beneficial is in smart assistive care facilities. Many societies face the problem of an aging population. The increase in the population of elderly people will place increasing pressure on existing nursing and care resources (Cheek et al., 2005). While technology shouldn't replace human care, smart home technology has the potential to improve the quality of life of people in need of support. Smart homes are living spaces designed with interactive technology, automation, and support systems that provide increased independence and wellbeing (Morris et al., 2013). Smart homes in assistive care contexts are typically equipped with a variety of sensors for analyzing the behavior of the occupants (Acampora et al., 2013).

As sensors, actuators and computing power become cheaper and more widely available, it is increasingly possible to equip elder care facilities with a smart infrastructure that can help residents achieve their daily tasks (Ranieri et al., 2021). Elder-care facilities could be equipped with a smart home architecture to ease the pressure on staff and improve the independence of residents. Alternatively, the homes of elderly people can be equipped with smart technology in order to facilitate aging-in-place. Many elderly people prefer to remain in their own homes as they age. Smart technology has the potential to improve the independence, safety, and health of elderly people living in their own homes. Smart home technology could aid elderly people with emergency assistance, fall detection, reminder systems, and assistance for those with hearing, vision, or cognitive impairments (Cheek et al., 2005).

Intention recognition is particularly beneficial in smart elder care facilities in a number of ways. First, it enables the smart home system to preemptively perform assistive actions and collaborate better with human occupants. For example, inferring the intention to go to bed can enable the system to preemptively close the blinds and switch off any lights in the house. Second, intention recognition can be integrated with a smart reminder system. Many elderly people live with some form of cognitive impairment. A smart reminder system helps elderly people feel more in control of their daily activities and prevents accidents (Demiris et al., 2004). Intention recognition can augment such a system by triggering reminders at the most opportune time. For example, on inferring the intention to go to bed, the system can trigger reminders to lock the doors and switch off the cooker. The system can remind the resident to take their appropriate medication on inferring the intention to eat dinner.

We implement an intention recognition system suitable for elder care scenarios based on the principles of statistical relational artificial intelligence (StarAI) (De Raedt et al., 2016), specifically, we use the language ProbLog (De Raedt et al., 2007). ProbLog is a probabilistic extension of Prolog that is situated within the StarAI paradigm (De Raedt et al., 2007). ProbLog programs look very much like Prolog programs, except that clauses can be labeled with the probability that they are true. ProbLog calculates the probability that a query is true. ProbLog can also estimate probability parameters. Given a program where some of the probability labels are missing, ProbLog can estimate the missing probabilities when given a set of observations. The combination of tools offered by ProbLog makes it a powerful tool for reasoning about agent's intentions. It can represent both uncertainty and logical relations.

Our research demonstrates how intent recognition can be achieved using ProbLog. We use ProbLog to build models that predict the intention of an agent given observations of the agent's actions as well as environmental factors such as temperature and time. We employ assisted living facilities as our motivating example. ProbLog can represent and reason over relational directed probabilistic graphs. In other words, ProbLog can represent individuals and the relations that exist between them, while also reasoning probabilistically. We use ProbLog to implement relational directed probabilistic graphs that represent the dependencies between environmental factors, intentions, and actions. Given an observation of an agent's actions and factors of the environment such as time, the network can be queried to find the most probable intention to have caused those actions.

The article is structured as follows: after introducing the problem in Section 1, we investigate existing literature in Section 2. Section 3 introduces our technique, which is evaluated in Section 4. In Section 5, we discuss the results and proposed future work. The final section summarizes our findings.

## 2. Related Work

There exist a variety of approaches to implementing intention recognition. In this section, we present some representative examples from across the spectrum and comment on their advantages and disadvantages.

Broadly, the techniques present in the literature can be put into three categories: logic based, probabilistic, and neural network based. Many logic based implementations rely on performing abductive inference given an observation of behavior and a plan library (Sadri, 2011). Informally, abductive inference is the process of generating the best explanation given background knowledge and an observation (Denecker and Kakas, 2002). For example, if an agent knows that *p*⇒*q* and the agent observes *q*, then the best explanation for *q* given the background knowledge is that *p* is true. In intention recognition, the system usually consists of the following components: background knowledge, knowledge about how plans and goals are related, a set of possible intentions, and an observed action sequence. The background knowledge is often a plan library (Sadri, 2011).

An example of a purely logical approach is an enemy intention recognition system from (Mulder and Voorbraak, 2003). Their framework allows for intention recognition even when observations of actions do not contain all of the relevant information, that is, when observations are of non-ground actions such as ∃*Xtakeoff*(*X, carrier*1). Their system relies on performing abduction given such observations and a plan library. Given a plan library and an observation, the system finds a set of hypotheses consistent with the library and the observation. Further examples of logic based approaches can be found in Myers and Lee (1997), Jarvis et al. (2005), and Sindlar et al. (2008).

There are also frameworks that combine logic and probability. An example from assisted living is a system created by Pereira and Han (2009). They use situation sensitive Causal Bayes Nets (CBNs) (Pearl, 2009), implemented in a declarative language called P-log, to model the intent recognition problem. The nodes in the network encode causes, intentions, and actions. The networks are implemented as facts and rules in P-log. Causes have either unconditional or situation sensitive probabilities. For example:


(1) $$\begin{array}{r} P\left(thirsty\right)is\;50/100 \end{array}$$

(2) $$\begin{array}{r} P\left(thirsty\right)is\;70/100\leftarrow temperature>30 \end{array}$$

Where equation 1 encodes that the probability of an agent being thirsty is 0.5, and Equation (2) encodes that the probability of the agent being thirsty *given* that the temperature is above 30 degrees is 0.7.

The probability of an intention given a cause, and the probability of actions given intentions are encoded as rules. for example:


(3) $$\begin{array}{r} P\left(intention\;to\;look\;for\;drink\right)\;is\;90/100\leftarrow thirsty \end{array}$$

(4) $$\begin{array}{c} \begin{array}{c} P(looking\;for\;something)\;is\;99/100\leftarrow intention\;to\;look\;for\;a\;book\\ \;is\;true\wedge intention\;to\;look\;for\;a\;drink\\ \;\;\;is\;true \end{array} \end{array}$$

Equation (3) expresses that the probability of the agent intending to get a drink is 0.9 if they are thirsty. Equation (4) means that the probability of the agent performing the action of looking for something is 0.99 if they intend to look for a book and intent to look for a drink.

In their implementation, all of the probabilities are predefined by the programmer. One of the key strengths of this system is the integration of causal environmental factors into the model. However, the representation of probabilistic information is far less straightforward in P-log than in ProbLog.

Logical frameworks can make use of relational reasoning, important in a world of individuals and relations between them. They can also be integrated with other logic based systems such as planners. A major drawback of logical frameworks is the difficulty of representing uncertainty. Traditional probabilistic approaches such as hidden Markov models, on the other hand, are able to represent uncertainty, but lack relational reasoning. Combining probabilistic and relational reasoning would clearly have major benefits. It is for this reason we have identified ProbLog as a powerful tool for intent recognition in smart homes.

Intention recognition can also be achieved using neural networks. A recent example is a system using recursive neural networks for plan recognition (Bisson et al., 2015). The authors use recursive neural networks to learn a decision model that infers an agent's plans *via* a plan library.

Such neural network frameworks are able to deal with uncertainty and incomplete knowledge, and often attain impressive levels of accuracy. They do, however, have some significant drawbacks. One common issue is the large amount of data needed for training. Such large amounts of data are costly to generate (Punjani and Abbeel, 2015; Pinto and Gupta, 2016; Levine et al., 2018). Some promising ways of dealing with this are emerging, such as using simulated data (Kappler et al., 2015), digitally manipulated data (Neverova et al., 2014), or crowd-sourced data (Pratt, 2015). But a further problem is that the kind of data that allows deep learning to excel is high-dimensional sensor data. This is often the kind of data we expect to see in robots, but is not necessarily the case in intention recognition. Further difficulties are the time needed to train a deep neural net (Pierson and Gashler, 2017) and that the amount of computational resources needed for neural networks are not always practical (Zhang et al., 2016).

Recent work explores using ProbLog for action recognition in smart homes (Sztyler et al., 2018), and using ProbLog for smart home control *via* probabilistic agents (Mekuria et al., 2019). There is no existing work on implementing intent recognition in smart homes with ProbLog^1^.

Markov logic networks, a statistical relational technique related to ProbLog, have been used for goal and plan recognition (Singla and Mooney, 2011; Ha et al., 2014). However, as emphasized by Sztyler et al. (2018) representation of probabilities is more straightforward in ProbLog. Further, unlike Markov logic networks, ProbLog makes a closed-world assumption, meaning that ProbLog requires less grounding operations and is therefore more scalable.

## 3. Intention Recognition in ProbLog

### 3.1. Background

In this section, we define the intent recognition problem and briefly introduce ProbLog.

#### 3.1.1. Intent Recognition

Intuitively, intent recognition aims to answer the question “why is an agent behaving this way?” (Freedman and Zilberstein, 2019). The answer to such a question is provided by inferring the agent's intention, where an intention is a high level cognitive desire to which an agent has committed to fulfilling (Wooldridge, 2003). A particular agent boils a kettle and opens a jar of coffee because that agent desires to drink coffee, and has committed to fulfilling this desire. Inferring an agent's intention depends on observations of that agent's behavior, observations of salient features of the environment, and background knowledge that encodes how these influence each other (Mirsky et al., 2021). Informally, then, in the process of intention recognition an observing agent attempts to infer the intentions of an observed agent by analyzing its behavior. An intention recognition problem consists of some possible intentions, observations of behavior, and observations of the environment. More formally, an intention recognition problem is defined as follows:

**Definition 1 (Observable actions)**. A is a set of symbols that represent the observable actions of the observed agent.

These symbols map to particular actions that the observed agent may perform in the environment. In many cases an agent may also perform actions that are not observable. These would not have any corresponding symbol in A. The symbols in A are simply labels for the actions observed, and have no further internal structure. If deployed in a real smart environment these symbols would need to be inferred from raw sensor data. This process is known as action or activity recognition (Mirsky et al., 2021). An action recognition system could be a novel system implemented in ProbLog, but it could also be an existing technique that takes as input raw sensor data and outputs symbolic tokens representing observed actions. In this article, we assume action recognition from low level sensor data and subsequent mapping to these symbols to be given. In a simple smart kitchen example, A could include switchKettle, getMug, and openFridge, encoding the behaviors of switching on a kettle, grabbing a mug or opening the refrigerator.

**Definition 2 (Observable environmental properties)**. E is a set of variables that represent properties of the environment that can be observed.

The variables in E are assigned values depending on sensor observations, where the raw observations are assumed to be binned into appropriate categories. For example, room temperature observations could be binned into either cold, comfortable, or hot.

**Definition 3 (Intentions)**. I is a set of symbols that represent the possible intentions of the observed agent.

In the smart kitchen example this might include iMakeCoffee, iMakeDinner, iWashDishes to encode the intention to make coffee, cook dinner, or wash the dishes.

Finally, we define an intent recognition problem as follows:

**Definition 4 (Intent recognition problem)**. An intent recognition problem is a tuple R=(I,A,E,O). I, A, and E are defined as above. O is a set of observations O=(V,F), where V is an observed sequence of actions $V=\left[a_{1},...,a_{n}\right]$ such that $a_{i}\in A$, and F is a set of observed environmental properties $F=\left[e_{1},...,e_{k}\right]$ such that $e_{i}\in E$.

Given an intent recognition problem, the task is to calculate the probability distribution over I given the observation O. The intent with the highest probability is the predicted intent attributed to the agent:


(5) $$\begin{array}{r} g=\arg\underset{I}{\max}P\left(I|O\right) \end{array}$$

In assisted living scenarios the problem is to infer the intention of the resident given observations of their behavior and of the environment. Examples of possible intents include the intention to prepare a meal or to watch television. Examples of observable actions might include turning on a light, opening a cupboard or sitting in a chair, while environmental observations are of properties such as temperature, humidity, and time.

Generally, intent recognition can fall into the following three categories (Mirsky et al., 2021):

- Keyhole recognition: the observed actor is not aware of being subject to intent recognition.
- Adversarial recognition: the observed actor may take action to obscure inference of it's intent.
- Intended recognition: the observed actor behaves such that it's intent is easier in infer.

We only consider keyhole recognition, since this is the most common in a smart assisted living context.

Finally, in an assisted living context an ideal intent recognition system would be capable of performing intent recognition on multiple agents, however in this article we only address intention recognition on a single agent.

#### 3.1.2. ProbLog

ProbLog is an extension of Prolog whereby clauses in the program can be annotated with mutually independent probabilities (De Raedt et al., 2007).

Prolog is a logic programming language particularly suited to for problems that include objects and relations between them (Bratko, 2001). Prolog programs consist of clauses. Clauses can be facts or rules. A query is a clause that encodes a question posed (Bratko, 2001).

Like Prolog, a ProbLog program consists of clauses, where each clause can be a fact or a rule. In ProbLog each clause can have an associated probability. The following example demonstrates a simple ProbLog program:


          0.3::thirsty.
          0.9::wantDrink :- thirsty.


The first clause expresses a probabilistic fact: that thirsty will be true with a probability of 0.3. The second clause is a rule indicating that if thirsty is true, then wantDrink will be true with a probability of 0.9.

Prolog computes the success or failure of a query. ProbLog, in contrast, computes the probability that a query succeeds. Below follows a summary of the equations for computing this probability, as summarized from De Raedt et al. (2007) and Sztyler et al. (2018).

A ProbLog program is defined by *T* = {*p*_1_:*c*_1_, ..., *p*_*n*_:*cn*}, where *c*_*i*_ is a clause and *p*_*i*_ is its associated probability. Given a program, the probability of a world *w* is defined as:


(6) $$\begin{array}{r} P\left(w|T\right)=\underset{c_{i}\in w}{\prod }p_{i}\underset{c_{i}\in w_{T}\backslash w}{\prod }\left(1-p_{i}\right) \end{array}$$

Where *w*_*T*_\*w* denotes the set of clauses that are in *T* but were not instanced in *w*, that is, the set of false ground probabilistic atoms. The probability that a query *q* succeeds in the program *T* is given by:


(7) $$P(q|w)=\{\begin{array}{ll} 1, & \text{if }\exists \Theta :w⊨q\Theta \\ 0, & \text{otherwise} \end{array}$$

(8) $$\begin{array}{r} P\left(q,w|T\right)=P\left(q|w\right).P\left(w|T\right) \end{array}$$

(9) $$\begin{array}{r} P\left(q|T\right)=\underset{w\subseteq W}{\sum }P\left(q,w|T\right) \end{array}$$

That is, the probability that a query in ProbLog succeeds is the sum of the probabilities of the worlds where *q* can succeed.

A detailed tutorial on how to use ProbLog can be found at https://dtai.cs.kuleuven.be/problog/tutorial.html.

### 3.2. Underlying Model

We use ProbLog to build directed probabilistic graph models (Tahboub, 2006). We implement a network that has three layers of nodes:

- The bottom nodes represent the set of observable actions. Every observable action is encoded as a node. There are *n* nodes in the bottom layer, where *n* is the number of observable actions in A. Each node can either take the value true or false.
- The middle nodes encode the set of intentions. There is one node for every intent *i* in I, that is, there are *j* nodes, where *j* is the number of possible intents in the set I. Each node is either true or false.
- The topmost nodes represent factors that influence the possible intentions. These can be predispositions, environmental factors, and other background factors. There are *k* nodes, where *k* is the number of observable environmental properties in E. The top nodes can take a number of values, depending on what they encode.

The edges in the graph represent causal dependence between nodes. The nodes in each layer are causally dependant on the nodes in the layer above. There should be an edge between nodes where the node in the upper layer causally influences the value of the node in the lower layer. Every intermediate node is accompanied by a specification of its conditional probability distribution given its parents in the graph. Top layer nodes are accompanied by a specification of their unconditional probability.

**Figure 2:**
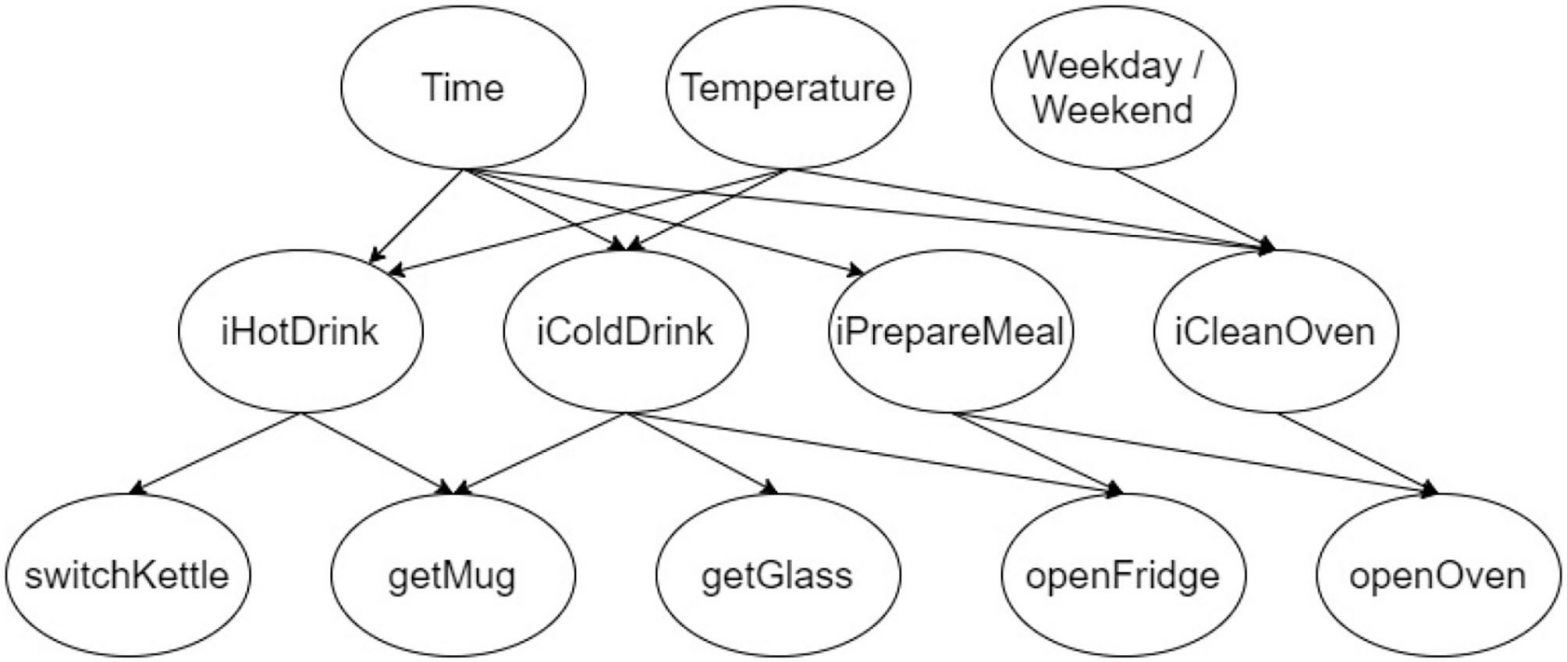
Kitchen example graph structure.

Figure 2 illustrates a portion of an example model for intent recognition in a smart kitchen. In this example model, the possible intentions include the intention to make a hot drink, to make a cold drink, to prepare a meal, and to clean the oven. Each possible intention has a corresponding node in the network.

The set of observable actions includes switching on the kettle, grabbing a mug or glass, and opening the fridge or oven. The edges from the middle nodes to the bottom nodes indicate causal connections between intents and actions. There is an edge between the nodes iHotDrink and getMug are connected because an agent's intention to make a hot drink is a causal factor in the production of the action of getting a mug. Wanting to make a drink does not cause an agent to open the oven, so iHotDrink and openOven are not connected.

Finally, the kitchen example includes the following environmental factors that influence the arising of an intent: temperature, time of day, and whether it is a weekday or a weekend. The edges from the top nodes to the intermediate nodes indicate which factors causally influence the probability of each intention.

After each new action observation, alongside observations of other influencing factors such as time and temperature, the probability of each possible intention in the model is queried. We take the intention with the highest probability to be the prediction. For example, if there are 4 possible intents, for all 4 we query the probability that it is the true intent, the one with the highest probability is the prediction.

### 3.3. Implementation in ProbLog

In this section, we explain how to implement the model introduced above using ProbLog. We describe in detail the steps required to take a graphical model like that introduced in the previous section and encode it as a ProbLog program. Briefly, these steps are as follows:

1. Define basic domain facts, for example the residents present in the home.
2. Encode the possible values of the influencing environmental factors as a series of probabilistic facts.
3. Encode the dependence of intentions on environmental factors as a series of probabilistic rules.
4. Encode the dependence of actions on intentions as a series of probabilistic rules.
5. Define queries (one for each intention).

The first step is to define basic domain facts in order to ground the rest of the clauses in the program. In the smart kitchen example, the later rules in the program take a resident as an argument. To ground the rest of these clauses we therefore have to define facts that encode those residents in the program. We encode the residents of the home in the program using ground atoms. These facts are defined in ProbLog as follows:


 
person(tom).
person(andy).
 


The graphical model itself is encoded in ProbLog as a series of probabilistic facts and rules. First, the nodes in the top layer are encoded as probabilistic facts. There is one node in the graph for each observable environmental factor E, and there will be one ProbLog fact for each node. The facts are encoded using ProbLog's; operator, which denotes the XOR operator. Only one of the atoms in the clause can be true, where each atom is annotated with the probability that it will be true. We assign each atom in the clause an equal prior probability, although in practice which one is true is observed and provided to the model as evidence. To illustrate this, the top layer nodes in the kitchen example are expressed as follows:


 
1/5::morning; 1/5::midday; 1/5::afternoon;
1/5::evening; 1/5::night.
1/3::tCold; 1/3::tComfortable; 1/3::tHot.
5/7::workday; 2/7::weekend.
 


The middle layer intent nodes are encoded as probabilistic rules with the intent as the rule head, and the factors that influence the intent as the tail. There is one node in the graph for each possible intent in I, and each node is encoded with a set of ProbLog rules. The set of rules for each node defines the probability distribution of that node given the environmental factors on which it depends. There is one rule for each binary parent node, and *n* rules for every non-binary parent node, where *n* is the number of possible values the parent node can take. Currently, probability labels are defined and tuned by a human expert.

To clarify, take the prepare meal intention in the kitchen example. If we assume that the only parent node of iPrepareMeal is time of day. Time of day can take 5 values, so there is one rule for each value:


 
0.5::iPrepareMeal(X) :- morning, person(X).
0.8::iPrepareMeal(X) :- midday, person(X).
0.2::iPrepareMeal(X) :- afternoon, person(X).
0.9::iPrepareMeal(X) :- evening, person(X).
0.01::iPrepareMeal(X) :- night, person(X).
 


The first rule expresses that there is a probability of 0.5 that X intends to prepare a meal, where it is the morning and X is a person in the home. Because residents are stated as ground facts in the domain, person(X) functions to ground the clause.

The action nodes are encoded in the same way. The set of rules for each action node represents the distribution of an action occurring given the intents on which it depends. Observing the refrigerator being opened, for example, depends on the resident either intending to get a cold drink or prepare a meal:


 
0.7::openFridge(X) :- iColdDrink(X).
0.8::openFridge(X) :- iPrepareMeal(X).
 


Action observations are encoded in ProbLog using the pre-defined evidence predicate. Our model presupposes that action recognition has already been performed, and the observed actions have been mapped to the set A. In a smart home setting, action recognition systems typically infer the actions being performed by the occupant in real time *via* data originating from sensors installed in the environment and wearable sensors. In the ProbLog model we assume this process is done, and only encode which actions in the set A have been observed. We use ProbLog's *evidence* predicate to achieve this.

Finally, the system queries the probability of every possible intent. The queries are encoded with the *query* predicate. For each query ProbLog calculates the probability that the clause within the query predicate is true. The intent with the highest posterior probability is taken as the prediction.


  
% Observations of influencing factors.
evidence(afternoon,true).
evidence(tCold,true).
  
% Observations of actions
evidence(switchKettle(tom), true).
evidence(getMug(tom), true).
  
% Queries
query(iHotDrink(X)).
query(iColdDrink(X)).
query(iPrepareMeal(X)).
query(iPrepareSnack(X)).
query(iCleanOven(X)).
 


The posterior probability of each intent is influenced by both the top nodes and the bottom nodes. Observations of the factors in the top nodes, such as temperature or time of day, determines the prior probability of each intent. A set of action observations then determines the Bayesian posterior probability of each intent. These two directions of influence together allow competing hypotheses to be resolved that would not be possible in many purely abductive intent recognition systems, or graphical models that don't have this layer of nodes. Consequently, the correct intent can be predicted earlier in the action sequence. To illustrate this, imagine Tom is thirsty in the afternoon and intends to drink a cold drink. An observation of Tom opening the refrigerator corresponds to the competing hypotheses of an intent to make a cold drink, prepare a meal, or get a snack. Without the influence of the top layer of nodes, there needs to be a further action observation to convincingly narrow the likely hypotheses. But in our model the observation that it is afternoon makes greatly reduces the probability that Tom intends to prepare a meal, effectively narrowing the number of likely hypotheses earlier in the action sequence.

To sum up, building an intention recognition model with our technique follows the following process:

1. Observe agent's behavior to discover links between environment, intentions, and actions.
2. Map out graphical model to define probabilistic influence. This step is primarily to clarify for the engineer how the environment, intentions, and actions are linked.
3. Define domain facts, for example the residents of the home.
4. Encode the environmental factors in E as a series of probabilistic rules.
5. Encode the dependence of the possible intentions in I on environmental factors as a series of probabilistic rules with the intent as the rule head and the environmental factor as the tail.
6. Encode the dependence of observable actions in A on the intentions as a series of probabilistic rules. The action forms the head of the rule, the intention forms the tail.
7. Define queries (one for each intention).

**Figure 3:**
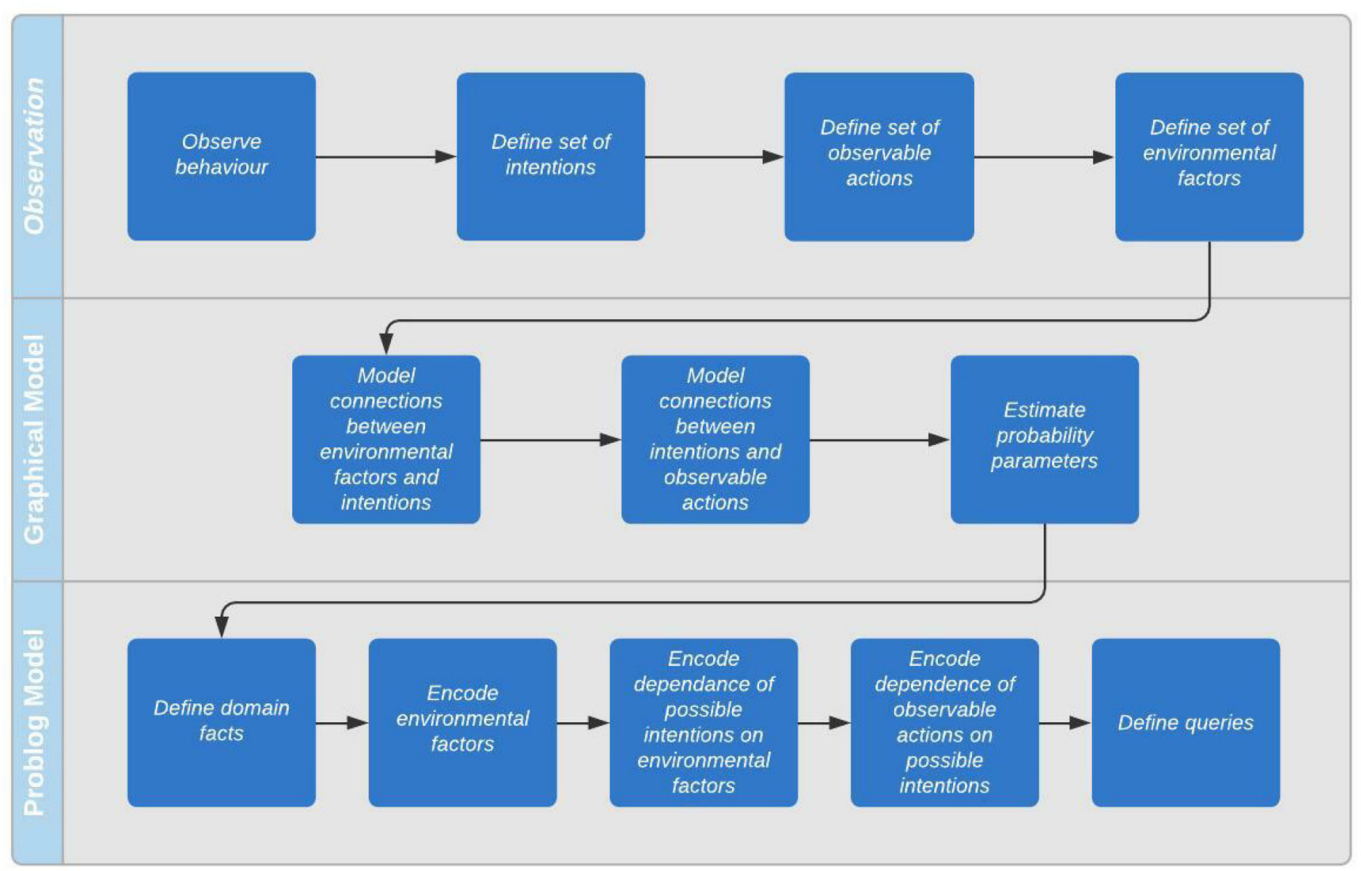
Model development process.

This process is illustrated in the flowchart in Figure 3.

## 4. Results

The main focus of our experiment is investigating how robust our technique is to reduced observability of the action sequence. Residents may perform actions that are not observable given the sensors installed in smart home. We used data generated from scripts to assess how robust our technique is to partial observability of the resident's action sequence.

Our experiment was performed with general smart home data sets, rather than data sets gathered specifically from smart elder care facilities. This choice is justified from an aging-in-place perspective, where the goal is to equip a patients own home with a smart architecture in order to extend the time that they can live independently in their own place. In this case, the smart facility is largely a typical smart home. Our approach is less indicative of a community elder care facility where many residents live together under the supervision of care staff. However, even in this case, the smart architecture is present largely to assist in everyday activities, and so is not too far removed from a smart home.

The experiment was run on a 2018 Dell XPS 15 running Windows 10 on a x64 Intel(R) Core(TM) i7-8750H 2.20Ghz 6 core CPU and 16GB of RAM.

The experiment models a smart home. Time of day is the only observable environmental factor in E and is divided into morning, midday, afternoon, evening. There are 8 possible intentions in I, and 27 observable actions in A. These include typical tasks and actions that an elderly resident may perform, such as getting a spoon and bowl to prepare breakfast.

To evaluate this model we used a script for generating plan recognition problems from Raḿırez and Geffner (2009). The inputs to the script are a domain description in the Planning Domain Definition Language (Malik et al., 1998), a set of possible goals *G*, and an actual goal *g* in *G*. The script outputs an observation sequence *O*. An observation sequence consists of a sequence of actions that achieves the goal *g*. The domain description consists of definitions of the possible actions that can be performed. The actions in the domain description correspond to the set of actions A in the intention recognition model. Similarly, the set of possible goals *G* inputted to the script corresponds to the set of intentions I in the intention recognition model.

To evaluate the effect of including background environmental knowledge in the model, we altered the script to generate the actual goal *g* with a probability dependent on the time of day. The value of the time of day was generated randomly. For example the script may randomly generate a time of *morning*. Given this time, the goal of making breakfast will be generated with a probability of 0.8. Given the goal to make breakfast, the script may generate an observation sequence such as: get cereal - get milk - get bowl.

The key aim of this experiment was to investigate the effect of reducing observability of the resident's actions on the prediction accuracy of the model. It may be that the facility is not equipped to recognize certain actions, for example, perhaps there is a sensor that detects when bowls are lifted but not spoons. Action recognition may fail for other reasons, a sensor could fail, for example. The upshot is that some of the actions in the sequence performed by the agent in fulfillment of the intention may not be observed. We evaluated with five levels of observability of the data: 100, 90, 80, 70, 60, and 50%. By observability we mean the percentage of actions in an action sequence that are actually observed. For example, at 90% observability an action sequence containing 10 actions will have one action randomly removed, meaning the observed sequence consists of 9 actions.

We evaluated using 500 examples. We performed three repeat experiments. Our evaluation metric is prediction accuracy, calculated by dividing the number of correct predictions by the total number of examples.The model prediction accuracy averaged over the three repeat experiments is presented and Table 1.

**Table 1:** Experiment 1 results.

**Observability (%)**	**Accuracy**
	
100	0.75
90	0.8
80	0.74
70	0.73
60	0.68
50	0.62

These results show that the prediction accuracy dropped by only 0.2 at 70% observability, dropping more sharply after. This indicates that even if only 70% of an agent's actions performed in pursuit of an intent are observed, the prediction accuracy will remain close to that at 100% observability.

An anomaly is present at 90% observability, where the prediction accuracy unexpectedly increases. This was present over multiple re-runs of our experiment. Observability is reduced by removing actions from the observation sequence. The actions are removed at random, so we are confident that this anomaly is not due to any pattern in removing actions from the observation sequence.

## 5. Discussion and Future Work

In this section we explore the upshot of our results, suggest some possible further examples, and indicate how our continuing research will expand on the results presented in this article.

### 5.1. Advantages and Disadvantages

Building intention recognition models in ProbLog has some important advantages, and a couple of drawbacks that need addressing.

An important advantage of implementing intention recognition models in ProbLog is the interpretability of ProbLog programs. Interpretability is a term that lacks a standard definition, but an interpretable AI system is widely accepted to mean a system whereby a user can inspect and understand mathematically how a model maps from inputs to outputs.

Certain limitations prevent AI from being routinely adopted in healthcare scenarios, these include the possibility of erroneous decisions, lack of clarity of liability when things go wrong, and difficulty in securing the trust of healthcare professionals and the general public. Building models that are intelligible to the engineers that build them and explainable to the healthcare professionals that use them is crucial to overcoming these limitations and advancing the deployment of AI powered technologies into healthcare environments. The above limitations also apply to intention recognition systems, especially in an assisted care context. As an example, suppose an intention recognition system underpins a reminder system. A resident must take a certain medication with a meal. The intention recognition system reminds the resident to take their medication when it infers the intent to eat dinner. If the system were to routinely incorrectly infer the intent to eat dinner, this would trigger reminders at the incorrect time, causing the resident to take their medication at the incorrect time. There is a possibility this could reduce effectiveness of their medication or cause other negative side effects. The result is harm to the resident. In this case how do healthcare professionals figure out what caused this result? Who (or what) is liable for the harm caused? How are residents to trust the system? Tackling these problems relies on engineers and healthcare workers being able to interpret and understand how the system made it's predictions, and explaining this process to end users.

The logical nature of ProbLog programs, coupled with the intuitive way in which probabilistic causes are labeled with probability parameters makes ProbLog programs highly interpretable. The mapping from the input to the ProbLog model to the output can be analyzed using mathematical logic and Bayesian mathematics. Traditional logical approaches to intention recognition are interpretable, but are less able to handle the probabilistic and noisy nature of the intention recognition problem. Other systems that combine logical and probabilistic reasoning, such as Markov logic networks (Ha et al., 2014), are mathematically interpretable, but are less intelligible than ProbLog due the unintuitive way in which probability distributions are defined in Markov networks (Sztyler et al., 2018). Deep learning approaches can achieve impressive levels of accuracy (Bisson et al., 2015), but are usually black box models that are not interpretable. On the whole, ProbLog is able to obtain reasonable prediction accuracy by reasoning logically and probabilistically, without sacrificing interpretability of intelligibility. The extent to which intention recognition models are intelligible and explainable is an empirical question. Conducting a study to establish this will be a component of our future work on this topic.

A significant disadvantage of our approach is that the model must be built by a human expert. At present, both the graph structure and the probability parameters must be hand coded. This requires a significant time investment from an engineer, which in turn is likely to increase the cost of the model for a user, and reduce the accessibility of the technique. Cost is a significant concern in the deployment of aging-in-place technology. Reducing the amount of time needed from human engineers is thus an important challenge to address in our future work. We have begun to tackle this with parameter estimation.

A further drawback of our current approach is that we don't take into account concurrent intentions. In many cases an agent may be pursuing multiple intentions at the same time. When this is the case it is likely that actions performed in pursuit of multiple intentions will be present in an observed action sequence. An intention recognition system would need to reason about multiple intentions at the same time and be capable of filtering out which actions in an observation sequence are in pursuit of the same intention. Our technique does not currently do this.

### 5.2. Relational Reasoning

Our examples so far have demonstrated probabilistic reasoning, but a key advantage of using ProbLog is that it can also perform relational reasoning. For example, we could change the iColdDrink predicate to take two take two arguments:


 
          0.6::iHotDrink(X, X) :- afternoon, tComfortable, person(X).
          0.7::iHotDrink(X, Y) :- afternoon, tComfortable, person(X),
                                  person(Y), \+identical(X,Y).
  
          0.9::grabMug(X, Y) :- iHotDrink(X, Y).
          0.3::grabMug(X, X) :- iHotDrink(X, Y).
          
 


This demonstrates ProbLog's relational reasoning capacity. Instead of just expressing that X intends to make a drink, we can express that X intents to make a drink *for* Y, where Y might be a another person or identical with X. Different clauses define different probability distributions in these cases.

The last two clauses state that there is a probability of 0.9 that X intends to make a hot drink for Y, when X grabs Y's mug, Where as there is only a 0.3 percent probability of the same intention when X grabs their own mug. What this means is that not only does observation that a mug has been picked up increases the posterior probability that iHotDrink is true, but the observation also allows ProbLog to reason about who X intends to make the drink for. The nature of ProbLog as a relational probabilistic language means models capable of this kind of reasoning can be implemented in a much more compact way than traditional probabilistic graphical models.

It's important to note that although the model references two agents, this is not multi-agent intention recognition since only the intentions of one agent are being inferred.

### 5.3. Parameter Learning

Our kitchen model achieved prediction accuracy in the range of 70% to 80%. Although the model remained robust when reducing observability, this level of accuracy means that it would be difficult to deploy the model in a real smart care facility. For certain low risk scenarios, such as informing reminders for locking doors, the current accuracy may not be a problem. But more high risk scenarios would present a problem. The main factor likely to be preventing our system from achieving higher prediction accuracy is that estimating the optimal probability labels is extremely difficult.

Defining the correct probabilities is a crucial and difficult part of the knowledge engineering stage of building our model. Defining the probability annotations correctly requires not only expert knowledge of the relations between the influencing factors, intentions, and produced behaviors of humans in general, but of individual residents within the home as well. The extent to which temperature influences the intent to prepare a hot drink or the preference for a snack at a certain time of day could be substantially different for each resident. Gathering the necessary expertise to manually program these probability distributions is unreasonable. ProbLog can learn the probabilities that annotate clauses from data with an expectation maximization based algorithm (details of the learning algorithm can be found in Gutmann et al., 2011). ProbLog's out of the box learning algorithms was too inefficient to learn the parameters in our large models, but we were able to learn the parameters for single rules. This is a proof of concept that supports further work in this area. Given the resource intensive nature of setting parameters, it is worthwhile investing more time to augment ProbLog's learning for use in this domain.

When learning the probability distributions from scratch, the clauses have annotations of the form t(_):


 
      t(_)::p_openFridge1.
      t(_)::p_openFridge2.
      t(_)::p_openFridge3.
  
      openFridge(X) :- iColdDrink(X, Y), p_openFridge1.
      openFridge(X) :- iPrepareMeal(X), p_openFridge2.
      openFridge(X) :- iPrepareSnack(X), p_openFridge3.
      
 


In this case, each probability is first initialized with a random value. When clauses are annotated with the form t(p), they are initialized with the value p.

The data consists of partial interpretations, that is, not every node is observed. Each interpretation should contain observations of every environmental node, the nodes corresponding to the observed sequence of actions, and the intent. Each interpretation is given in the evidence() format and is separated by lines of “-”:


 
      evidence(weekday,true).
      evidence(afternoon,true).
      evidence(tHot,true).
      evidence(openFridge(tom),true).
      evidence(iColdDrink(tom),true).
      -----
      evidence(weekend,true).
      evidence(evening,true).
      evidence(tComfortable,true).
      evidence(openFridge(tom),true).
      evidence(iPrepareMeal(tom),true).
  
      
 


To sum up, although the graph structure would still need to be designed by an expert, the laborious task of assigning probabilities to each clause could be learned from an intent recognition data set. As a proof of concept we were able to learn single rules. Future work will focus on scaling this to learn all the parameters in an intent recognition model. We hope incorporating learning will also improve the prediction accuracy of the technique.

## 6. Summary

In this article, we have demonstrated how the probabilistic logic programming language ProbLog can be used for intent recognition. We have put forward elder care scenarios as a motivating example and explained the advantages of using ProbLog for intention recognition in elder-care. We have shown that ProbLog can capture the dependencies between environmental factors, intentions, and actions to build a model capable of inferring the intentions of smart home residents. Two key advantages of ProbLog are that ProbLog programs are highly interpretable and background knowledge can be easily included in the model. This enables the system to be effectively personalized for individuals.

We have empirically evaluated our model using a domain modeling a smart home. Our model achieved reasonable accuracy in this domain. Importantly, our technique remained robust even when actions were only partially observable. This domain is a simplification of the real smart assisted living environments in which we intend the models to be used. Therefore, we plan to use participants' interactions with a smart home facility to further evaluate the model.

ProbLog is capable of representing dynamic probabilistic graphical models such as dynamic Bayesian networks and hidden Markov models. We plan to incorporate this capability into our model to capture the dependencies of intents and actions on previous time steps. We also plan to explore using ProbLog's parameter learning capability to automatically learn the probability labels and reduce the burden on the human engineer.

## Data Availability Statement

The datasets presented in this study can be found in online repositories. The names of the repository/repositories and accession number(s) can be found below: https://github.com/shijiaruigbs/IntentionRecognitionProblog.

## Author Contributions

GS was responsible for developing the technique, designing, and conducting the experiments and writing up the results. RP and VB provided guidance and advice throughout the project and contributed to editing the manuscript. All authors contributed to the article and approved the submitted version.

## Conflict of Interest

The authors declare that the research was conducted in the absence of any commercial or financial relationships that could be construed as a potential conflict of interest.

## Publisher's Note

All claims expressed in this article are solely those of the authors and do not necessarily represent those of their affiliated organizations, or those of the publisher, the editors and the reviewers. Any product that may be evaluated in this article, or claim that may be made by its manufacturer, is not guaranteed or endorsed by the publisher.

## Footnotes

^1^During the write up and submission process an independent study was published (Acciaro et al., 2021).
